# Raising awareness of antimicrobial resistance among the general public in the UK: the role of public engagement activities

**DOI:** 10.1093/jacamr/dlaa012

**Published:** 2020-03-10

**Authors:** James Redfern, Laura Bowater, Lisa Coulthwaite, Joanna Verran

**Affiliations:** 1 Department of Natural Sciences, Manchester Metropolitan University, Manchester, UK; 2 Faculty of Medicine and Health Sciences, University of East Anglia, Norwich, UK; 3 Department of Life Sciences, Manchester Metropolitan University, Manchester, UK

## Abstract

In response to the accepted risk of emerging antimicrobial resistance, many organizations and institutions have developed and delivered events and activities designed to raise awareness of the issue and to change the behaviour of the intended audience. However, few of these events for a general public audience are documented or able to be sourced by those who might wish to repeat, adapt or modify, particularly those events that are successful. ‘Insider knowledge’ appears to be the best search tool. Moreover, evaluation of the success or impact of the event is rarely published. It would be useful if there were a ‘hub’ where descriptions of such activities could be deposited, enabling the building of a significant resource with real academic value.

## Introduction

In January 2019, the UK Government published its 20 year vision for antimicrobial resistance (AMR),^1^ stating that ‘By 2040, our vision is of a world in which antimicrobial resistance is effectively contained, controlled and mitigated’. The report, built on previous documents produced and commissioned within the UK^2–4^ recognizes the need to work with a variety of key stakeholders, outlining nine ‘ambitions for change’. The ninth ambition is specifically aimed at engaging the public on AMR. Although there is clearly significant international collaboration around AMR, our paper focuses on efforts made in the UK to address AMR through public engagement events aimed at the general public. We focus on the UK only because, as UK-based microbiologists and public engagement practitioners, we are aware of large numbers of activities that have taken place. We believe this to also be the case in other countries and hope that this paper will encourage similar endeavours elsewhere. Thus we have attempted to provide an overview of the range of activities that have been implemented by selecting examples that represent different approaches/audiences, and which are citable, in order to facilitate searches made by others.

## Methods

This is not a systematic review of the literature on public engagement with AMR (in part because so few activities have been published). Rather, we had to rely on personal experience and knowledge of activities delivered by colleagues and their contacts. Our single criterion for inclusion in this review was that the activity was searchable. In order to widen our search, 25 UK-based academics, artists and organizations known to the authors for AMR-related activities were directly approached (by e-mail), asking for citable examples of their relevant work. Seventeen responded [6 (of 7) organizations, 3 (of 6) artists and 8 (of 12) academics], of whom 11 provided examples that are cited in this paper. Additionally, indirect evidence of events was sought via social media. Our findings are presented in the Results sections. They are divided into two sections, the first focusing on direct messages to the public from health professionals and scientists. The second describes a variety of cross-disciplinary collaborations, primarily involving arts and sciences, where AMR is addressed as part of the output.

### ScienceDirect document search

In order to gain insight into the number of publications relating to the public engagement of AMR, a document search using the ScienceDirect search tool (https://www.sciencedirect.com/) using pre-defined search terms was carried out. Search terms were required to feature in the ‘title, abstract or author-specified keywords’ to be included. Search terms can be found in Table 1.

**Table 1.** Number of documents returned using specified search terms using the ScienceDirect document search tool

Search terms	Total	Peer-reviewed papers	Magazine articles
‘Antimicrobial resistance’ + ‘outreach’	0	0	0
‘Antimicrobial resistance’ + ‘public engagement’	6	5^28^^,^^29^^,^^53–55^	1^56^
‘Antimicrobial resistance’ + ‘public event’	2	2^57^^,^^58^	0
‘Antibiotic resistance’ + ‘outreach’	3	2^59^^,^^60^	1^61^
‘Antibiotic resistance’ + ‘public engagement’	2	2^62^^,^^63^	0
‘Antibiotic resistance’ + ‘public event’	0	0	0
Total	13	11	2

### Ethics

Since this is a review of citable output by others, the ethics will have been considered previously by those authors. The only novel public engagement event presented is the event at Manchester Police Museum, found in the Collections section of this review. For this, ethical consideration was undertaken and approved by Manchester Metropolitan University, UK.

## Results

### Direct messages to the public from health professionals and scientists

#### Public health campaigns and national initiatives

Of course, large-scale campaigns to highlight issues of antibiotic resistance are not new. Studies have suggested that media campaigns are more effective than healthcare professionals at raising awareness of antibiotics, whilst healthcare professionals are more successful at changing behaviour,^5^ perhaps due to the perception of expert knowledge and a more direct contact between expert and audience. Large-scale campaigns have been supported by international bodies, for example the ECDC and the WHO.^6–8^

European Antibiotic Awareness Day (EAAD) was launched in November 2008. England and Scotland participated, using an existing antibiotic awareness campaign that had previously run in February 2008. An evaluation of the November campaign (using a large ‘before and after’ public survey) found that whilst 83% of participants were potentially exposed to the campaign, there was no positive effect detected in terms of understanding the relationship between antibiotics and viral infection or reported use of antibiotics.^9^ Nevertheless, the EAAD has continued and is still supported by EU member states. In a 5 year perspective on EAAD produced by the ECDC, a decrease in the amount of antibiotics being consumed by the European population was noted, as well as an increase in understanding that antibiotics do not kill viruses, indicating that the efforts of EAAD provide valued support alongside member state national campaigns.^10^ In 2015, the WHO launched World Antibiotic Awareness Week (WAAW), alongside its Global Action Plan to tackle AMR, which the UK is committed to supporting,^11^ with the WAAW now encompassing EAAD. International campaigns such as these bring focus and global geopolitical support to campaigns operating on a national level. In the UK, there are various national campaigns designed to raise awareness of, and change attitudes towards, AMR. For example, Antibiotic Guardians (https://antibioticguardian.com/), led by PHE, asks people to pledge towards a series of behaviour changes around antibiotic prescribing, use and stewardship, infection prevention and control, self-care and health-seeking. The website has been heavily used (visited almost 500 000 times up to the end of 2017),^12^ with pledges from 129 countries, increasing year on year. Translations are enabling a worldwide awareness campaign.

An early evaluation of the programme suggests that while 69% of pledges came from healthcare professionals, and the remaining 31% from members of the public,^13^ it was not possible to demonstrate that this had led to a change of behaviour or long-lasting knowledge. Other national campaigns include Antibiotic Action (http://antibiotic-action.com/), which was launched in 2011 and was wholly funded by BSAC, producing a range of resources designed to inform different stakeholders, from politicians to members of the public. A well-publicized national campaign was launched in 2017, aimed at reducing patient expectation for antibiotics and supporting a reduction in prescriptions^12^ to complement the government’s ambition to ‘halve inappropriate prescribing of antibiotics by 2021’.

The Longitude Prize reflects the national priority given to AMR. It is an international prize fund (£10 million) led by the UK-based challenge innovation foundation Nesta (www.nesta.org.uk). In 2013, a public vote in the UK selected AMR as the global challenge for which the prize should be awarded (against the challenges of dementia, paralysis, sustainable food and clean water and eco-friendly flight) with a goal to develop a transformative point-of-care test that is useable anywhere in the world to ensure correct and appropriate prescription of antibiotics (https://longitudeprize.org/antimicrobial-resistance). The public vote was launched with a special episode of Horizon, a long-standing BBC science documentary series, and announced 1 month later live on the prime-time BBC magazine-style The One Show. Whilst this exposure and encouragement of public involvement is likely to have engaged large numbers, no literature could be found that evaluates the impact the choice of AMR as the winning topic has had on public understanding and/or perception of the problem.

#### Digital and social media

Many individuals or organizations interested in discussing AMR with the public have used digital/social media to good effect. For example, The Conversation (www.theconversation.com) is an independent online news service, where academics write topical articles in a journalistic manner, which can then be accessed and reproduced by more than 22 000 websites with potential reach of tens of millions of people.^14^ At the time of writing, a search of key terms revealed that 52 articles have been published on the topic of AMR, helping to ensure that academic research is translated into widely accessed online media. Other social media outlets, for example Twitter, are welcomed by professionals engaged with the public conversation of AMR.^15^ The hashtag #antimicrobialresistance was featured in over 46 000 tweets over a year (2015–16).^16^ Predominantly these tweets were from accounts based in the USA and the UK and usage spiked when an associated news event occurred (e.g. the banning of antibiotics from farming in the EU). The biggest ‘influencers’ in this analysis were considered to be international/national agencies including @CDCgov, @PHE_uk and @EU_Health.^16^ Other analysis of the BSAC Twitter account showed @TheUrgentNeed had 9000 followers from over 130 countries, whilst the South African Antibiotic Stewardship Programme (@Southafricanasp) engaged with followers in 82 countries.^15^ Data from the social media analytics tool keyhole.io show that over the 5 day period between 6 and 10 July 2019, the hashtag #antimicrobialresistance featured in approximately 197 posts, reaching an estimated 293 388 people, being viewed over 460 000 times. However, whilst the potential reach of social media is enticing, it is often exceptionally difficult to gauge success/understanding of tweeted messages and the audience will often be limited to those who follow a particular handle and/or hashtag, potentially an already engaged and/or scientifically literate population.

The e-Bug programme is led by PHE, in cooperation with a consortium of 28 international partner countries. e-Bug aims to educate children and young people about microbiology, hygiene and the spread and treatment of disease. In order to reduce the incidence of antibiotic resistance, e-Bug also aims to reinforce awareness of the benefits of prudent antibiotic use and the impact of inappropriate use.^17–19^ e-Bug provides free educational games and teaching resources, for both primary and secondary schools, and community activities (https://e-bug.eu). These e-Bug resources are recommended by NICE for all schools to teach about hygiene, infections and antibiotics.^20^ The PHE e-Bug team delivers ‘Train the Trainer’ workshops to encourage greater use of the resources with schools and community groups. e-Bug resources have been adapted with the addition of games and other activities for use as challenge badges by numerous Girl Guiding and Scouts groups in the UK and e-Bug has been working with members from Girl Guiding UK and Scouts UK to develop an Antimicrobial Resistance programme and badge. An evaluation of e-Bug’s junior and senior school programme was published in 2010,^21^ demonstrating that the teaching pack provided a significant improvement in junior student knowledge in all sections supported by e-Bug, that was retained for at least 6 weeks. The senior school programme had inconsistent results, varying between regions, but consistent improvement was observed in the UK cohort. Another evaluation of e-Bug used in a public engagement setting demonstrated improved knowledge of antibiotics in both adults and children.^18^

The Game Doctor (https://gamedrlimited.com/about/) is an organization aimed at developing digital education/training solutions in the healthcare-related sector, including games related to AMR such as the Bacteria Combat game, available on Android and iOS, developed in collaboration with the University of Glasgow. Evaluation of Bacteria Combat, which included a mixed-methods approach of both qualitative and quantitative analysis, demonstrated a significant proportion (95%) of school students (aged 9–12, *n *=* *150) found the game interesting. Further pilot trials demonstrated greater potential for engagement with multiplayer variants of the game.

#### Hands-on/citizen science

Antibiotics Unearthed was a national campaign operated by the UK-based Microbiology Society (https://microbiologysociety.org/education-outreach/antibiotics-unearthed.html), launched following the success of the Small World Initiative (SWI), a USA college-level engagement programme. The aim of the SWI was to bring authentic research to schoolchildren to discover potential new antibiotics via culturing of soil and has been considered a success by educators.^22^^,^^23^ However, Antibiotic Unearthed not only targeted those in education (both school and university) but also members of the public, using a citizen science approach via a series of pop-up events across the UK and Ireland. Members of the public were encouraged to take part in a variety of hands-on activities, culminating in the collection and preparation of soil to be analysed for potential new antibiotic compounds. Participants were able to track their samples and subsequent analysis online following the event.

Another citizen science-type activity is Swab and Send, initiated by UK academic Adam Roberts (https://www.lstmed.ac.uk/public-engagement/swab-send). The programme, started in 2014,^24^ asks members of the public to pledge £30 in return for five swabs for them to sample a location of their choice. Once the swabs are returned, they are analysed for microbial species, which may produce novel antimicrobial compounds. The programme has also been delivered using other mechanisms, for example at the Science Museum adult-only evening event Science Lates. Data published on the Swab and Send Facebook page (https://www.facebook.com/swabandsend/) show a wide geographical distribution, with participants in Scotland, England and Wales. The programme also aims to use research projects to engage primary and secondary school students with the challenges of AMR.^12^ The Swab and Send project has also generated research outputs detailing properties of potential pathogenic resistant isolates.^25^ An obvious extension from the hands-on activities often used to isolate bacteria is ‘agar art’, where the inoculum is placed on the agar so as to encourage a more aesthetic appearance of microbial growth: this has been used to effect for AMR (http://www.ox.ac.uk/news/2015-09-25-microbe-artwork-shows-limits-antibiotics).

The Seriously campaign was launched in Leeds in 2016 to educate university students on the importance of correct use of antibiotics and linking with Antibiotic Guardians (https://seriouslyresistant.com). This has expanded to citywide community events, public media videos, outreach to schools, support from pharmacies across Leeds and national campaign launches. The Seriously campaign has over 18 000 pledges, as of August 2019.

Many academics will have delivered local events to the public using similar principles. At one event in Manchester, UK, event organizers developed ‘a spoonful of soil’, comprising five sequential activities that engaged over 300 visitors in a 6 h period.^26^ Whilst the authors discuss the success of the event, they note the difficulty in evaluating participant knowledge/learning (especially long term) from an event such as this and suggest the use of mixed-methods evaluations (qualitative and quantitative analysis) and the addition of comprehensively trained evaluators at each event with the sole purpose of performing evaluation. Apart from this study, few papers could be found that reported on the success or otherwise of public awareness activities and campaigns designed to raise awareness about AMR, although several professional society magazines and university websites provide some narrative.

Museums and other venues such as town halls, arts centres and parks are often focal points for festivals, encouraging interdisciplinary events in order to attract different audiences. Many examples of AMR activities at festivals have already been noted above, but one recent example of immersive and cross-disciplinary outputs has been the gut microbiome and links of the human microbiome to AMR. The University of Salford’s MicrobiHome (https://scicomm.space/microbihome) and the Quadram Institute’s Guardians of the Gut (https://quadram.ac.uk/meet-the-guardians-of-the-gut) both allow walk-through immersive experiences.

### Cross-disciplinary collaborations

There are also opportunities for members of the public to engage with AMR where non-scientific subjects provide an alternative viewpoint or output. A number of funding bodies also recognized the value of multi-disciplinary approaches to AMR and crossover ‘sci–art’ collaborations, notably Wellcome through its AMR programmes and public engagement funds (https://wellcome.ac.uk/funding/schemes/public-engagement-fund). The Engineering and Physical Sciences Research Council (EPSRC) 2016 Bridging the Gaps AMR challenge (https://epsrc.ukri.org/funding/calls/bridgingthegapsepsamr/) enabled multidisciplinary approaches to AMR, resulting in a range of innovative and engaging interdisciplinary activities as part of the work package outputs.

In this paper, we have categorized cross-disciplinary sci–art outputs separately, utilizing the categories identified by the Arts Council (www.artscouncil.org.uk/what-we-do) for convenience: visual arts; collections; combined arts; dance; libraries; literature; museums; music; and theatre. The categories either address the creative discipline itself (art, dance, music, theatre or literature) or the vehicle through which creativity is demonstrated [collections, combined arts (festivals, arts centres etc.), libraries or museums]. Surprisingly or otherwise, we have identified activities designed to raise awareness of AMR in all of these categories, usually involving cross-disciplinary collaborations that might increase/provoke/inform the audience, and we have selected examples that illustrate the breadth of creative efforts focused on AMR. To simplify, we have combined categories and describe examples from the following: performance, visual arts, collections and literature.

#### Performance

Perhaps the most obvious type of performance for addressing AMR would be theatre. Productions tend to be immersive or interactive, encouraging audience participation.

##### Stopping the Spread of Superbugs

In 2010, the Microbiology Society presented *Stopping the Spread of Superbugs* at the Cheltenham Science Festival through the dialogue between two hospital cleaners. The performance was facilitated by a microbiologist who led discussion with an expert panel and the audience at key points in the story. The story reveals the fears and concerns of one of the cleaners after her mother is re-admitted to hospital with an infection following routine surgery. The audience was invited to put themselves in the place of the hospital decision-makers to answer some of the questions they face on a daily basis: should all hospital patients be pre-screened for superbugs on admission? Should antibiotics be used as a precautionary measure? How do infections arise at all if strict protocols are followed? The drama, featured as one of the top five things to see at the Cheltenham Science Festival, was a popular event and positive informal verbal feedback was gained from the audience,^27^ but the resource is no longer available.

##### The Drugs Don’t Work

Using a similar format, in 2017 a play entitled *The Drugs Don’t Work* was written, produced and presented in a collaboration between the Hobgoblin Theatre Company and Aston University, at the Cheltenham Science Festival and Birmingham’s Think Tank Science Museum. It focused on a pop singer who developed a sore throat, and addressed several misconceptions about antibiotic use and effectiveness. A pre- and post-performance questionnaire issued to the audience revealed improved audience knowledge, understanding and expectations (https://youtu.be/qL32XmoqfvU, https://youtu.be/2dMJIm3Qwqg).^28^

##### The Mould that Changed the World

*The Mould that Changed the World* (http://mouldthatchangedtheworld.com/) is a musical produced in partnership between BSAC and the Charades Musical Company, in collaboration with the University of Edinburgh, and launched in 2018. It narrates the story of antibiotic discovery, the rise of antibiotic resistance and the importance of preserving antibiotics for now and the future. Local scientists take part in the production and schools are encouraged to develop their own musicals using a resource kit (evaluation out soon, next phase of project also planned). A pre- and post-intervention survey was carried out in two schools and demonstrated that children had gained and retained knowledge 2 weeks following the musical.^29^

##### Nosocomial

*Nosocomial* ^30^ is a work in progress that focuses on healthcare professionals and the non-linear, almost performative nature of their work, intending to raise awareness of the science taking place behind the scenes, including AMR.

##### Artibiotics

*Artibiotics* (https://www.plymouth.ac.uk/news/musical-molecules-and-the-battle-for-biological-supremacy-laid-bare-in-new-performance) describes the battle between bacteria and antibiotics through music composed by Eduardo Miranda, Director of the Peninsula Arts Contemporary Music Festival, where the piece was performed in 2018. In the musical narrative, bacteria and antibiotics are presented as sound and the performance charts their quest to respectively damage and defend the DNA of their host. The project was commissioned by the research company Biofaction, and Miranda spent a period as artist-in-residence of the Synpeptide project, working alongside scientists at the Institute of Medical Microbiology and Hygiene in Regensburg, Germany. No information was available with regard to impact and evaluation.

Continuing the musical theme, the PHE provided another musical angle with its jingle (https://www.campaignlive.co.uk/article/public-health-englands-catchy-jingle-aims-danger-antibiotic-resistance-unforgettable/1447966).

##### Antibiotic Apocalypse

To engage young adults (aged 18–24 years) with AMR, Game Doctor (https://gamedrlimited.com/) developed an innovative short film, *Antibiotic Apocalypse*, that uses dance and emotive storytelling. A collaborative venture with the University of Glasgow and the Liverpool School of Tropical Medicine, an Edinburgh-based choreographer was recruited to oversee dance production, developing the production alongside specific learning outcomes. *Antibiotic Apocalypse* was released on YouTube, Vimeo and social media platforms on Hand Hygiene Day 2017, was entered into several competitions and film festivals and screened across the UK, including in patient waiting rooms. The planning of the film, its release and evaluation were thorough and findings were informative.^31^

##### Television and film

Two films, *Resistance* (full-length, directed by Michael Graziano, 2014) and *Catch* (short film, directed by Paul Cooke, 2017), similarly raise awareness of AMR. *Resistance* is a documentary outlining difficulties of controlling AMR, particularly due to issues with the use of antibiotics for livestock. The Society for Applied Microbiology (www.sfam.org.uk) sponsored a screening of the film with a panel of experts available to answer questions raised by the audience. Unfortunately, there is no documentary evidence of the event (which was well attended) and no evidence could be found for any impact of the film on audiences. *Catch* is more obviously designed to raise awareness of a post-antibiotic era and is eminently suitable for complementary discussion. The film was written and directed by Paul Cooke and Dominic Rees-Roberts, participants of the Wellcome Trust’s Emerging Talent programme for science communicators, and is backed by the Royal College of Pathologists and leading scientists from the University College London (UCL) Centre for Clinical Microbiology, the University of Cape Town, the University of Birmingham and Antibiotic Action.

There have been several television and radio programmes focusing on AMR, either as documentary programmes or drama. BBC Radio 4 has recently broadcast two radio series: *Resistance*,^32^ where a resistant bacterium causes an apocalyptic pandemic initially spread through ingestion; and *The Truth about Hawaii*,^33^ which focuses on one child battling a serious infection. Michael Mosley presented an informative television programme, *Michael Mosley versus the Superbugs* (https://www.renegadepictures.co.uk/program/michael-mosley-vs-the-superbugs_1487.aspx), in which he worked with artist Mellissa Fisher to demonstrate how the skin microbiome could acquire resistance using a human-sized agar sculpture, ‘microbial Michael’ (www.mellissafisher.com). Similarly, Angela Rippon’s *The* *Truth About Antibiotics*^34^ presented her investigations regarding AMR and potential alternatives.

In short, there is a relatively large and well-documented fund of performance and media outputs in existence that could be used to encourage engagement with AMR amongst a range of audiences, yet there is perhaps a lack of information on facilitative learning materials that might enable professionals to use them effectively.

#### Visual arts

There are a number of artists who use microbiology within their practice and several of them have focused on AMR, either independently or as an artist-in-residence. For example, Anna Dumitriu (annadumitriu.tumblr.com) has completed an impressive number of works, many of which touch upon AMR (for example https://labiotech.eu/bioart/anna-dumitriu-crispr-antibiotic-resistance/ and https://labiotech.eu/bioart/bioart-london-anna-dumitriu/). In one project, she worked with a team of scientists to help create a method to safely display a collection of ‘superbugs’ commissioned for the Science Museum London’s major exhibition ‘Superbugs: the Fight for Our Lives’. The exhibit was on show between November 2017 and April 2019 and was viewed by over 1.5 million visitors. A review^35^ provides an informative description of the entire exhibition. Along with Mellissa Fisher and Sarah Craske (sarahcraske.co.uk) and other ‘bio-artists’, Dumitriu uses art to provoke, engage and inform observers. Luke Jerram (Lukejerram.com) produces exquisite glass models of microorganisms, enabling us to contrast their beauty with the damage that they can wreak. The *SAW Antibiotics*^36^ publication gives an engaging overview of links between art, poetry, research and education carried out at Norwich Research Park and is, to our knowledge, unique as an accessible publication in the field.

#### Collections

Museums provide wonderful homes for public engagement activities. In addition to the Science Museum London ‘Superbugs’ exhibition noted above, the Eden project has hosted ‘Invisible Me’ and ‘Invisible Worlds’, both of which focus on microorganisms and, inevitably, give a mention to AMR. Specialist museums provide a more particular location, enabling customized activities. For example, the Stockport Tunnels, used as air-raid shelters in World War 2, hosted an evening event for adults.^37^ ‘Science in the Tunnels’ used parts of the shelters to engage the audience with aspects of AMR: the nurses’ room for storytelling about the pre-antibiotic era and the advent of the war; the food store for swabbing the tunnels for microorganisms in the hope of discovering a new antibiotic producer; and the blackout room for demonstrating some of the novel alternatives to antibiotics (using fluorescent-labelled cultures). Learning from a previous event,^26^ the aims were carefully specified and in addition to a post-event questionnaire, engagement was assessed through quantitative measures (number of agar plates used, number of hits on Flickr to see the results of plates post-incubation, number of contributions to a body map ‘where have you had infections for which you were given antibiotics’) and qualitative observation (of engagement and by noting questions asked during the evening^26^^,^^37^). This small event was therefore tailored to the venue and the audience and all evaluative indicators demonstrated that objectives were achieved, in the short term at least.

A subsequent event was hosted by the same team at the courtroom in the Manchester Police Museum. ‘Science on Trial’ utilized experts who spoke for and against the motion that ‘scientists will come up with a solution to AMR’. Again, qualitative observation of engagement was made. Before the event and after questioning by legal representatives (from the University Law Department), the audience (jury) were asked to vote on the motion. Interestingly, the number of respondents who believed in a solution fell after the event, perhaps reflecting that a more informed—and less optimistic—opinion had been made. Jury engagement related to the talent of the expert witnesses rather than to the content.

#### Literature

There have been several popular science books published about AMR (for example Bowater, 2017),^38^ but there is also a significant fiction resource that can be used to engage audiences with microbiology. The Bad Bugs Bookclub^39^^,^^40^ (https://www2.mmu.ac.uk/engage/what-we-do/bad-bugs-bookclub/) comprises scientists and non-scientists who read novels in which infectious disease forms part of the plot. Reading guides and meeting reports are provided for all of the 60 books so far discussed by the group. There are relatively few novels that feature AMR, perhaps because the infections tend to affect individuals rather than populations. Some rely on a terrorism-type activity where an antibiotic-resistant bacterium is deliberately introduced into a clinical setting (e.g. Cook, 1996).^41^ Some are adventure novels where the protagonists search for a new antimicrobial (for example Tabor, 2013).^42^ The graphic novel *Surgeon X*,^43^ published with accompanying learning materials and activities, attempts to engage audiences in discussion, and behaviour change, about a future without antibiotics. *Infectious Futures*^44^ is a series of short stories, commissioned by Nesta, addressing a future where AMR is commonplace. *A Fierce Radiance*^45^ is perhaps the only novel the book club has as yet encountered that addresses AMR in a factual manner but a fictional context. Set at the end of World War 2, at the beginning of the antibiotic era, it describes initial attempts to mass produce antibiotics and decisions as to who should be the first recipients of these drugs. We are made aware of the impact that antibiotics would have on many commonplace infections that affected people at that time, thus the impact of AMR is addressed indirectly. The book ambitiously encompasses romance, war, espionage, murder and history, but provides a good platform for discussion.

## Concluding comments

It is widely recognized that AMR is an issue that will critically affect future health and there are many schemes, programmes, initiatives and events taking place that are designed to raise awareness amongst the intended audience and change their behaviour. Implicit in all of these activities should be the identification of the key hypothesis/message and the particular audience, the design and delivery of the activity, and an evaluation of its success. Initiatives like the Antibiotic Guardian campaign have identified specific messages that enable activities to be tailored to fulfil aims. Interestingly, the International Scientific Forum on Home Hygiene (www.ifh-homehygiene.org) has recently published its own policy document (http://www.ifh-homehygiene.org/review-best-practice/hygiene-hypothesis-and-its-implications-home-hygiene-lifestyle-and-public), where they emphasize the importance of, and differentiation between, hygiene and cleanliness.^46^ They call for an integrated strategy for hygiene behaviour change in the home in everyday life, but as yet have not suggested the means by which their messages might be conveyed to audiences. This important link between hand hygiene, infection control and AMR^47^^,^^48^ could be built upon with appropriate activities devised and disseminated. An alternative approach to engaging audiences with issues around AMR might be through storytelling of experiences, humanizing clinical case studies, emphasizing the immediacy of AMR and encouraging discussion.^49^ The short film *Catch*, the movie *Resistance*, the *Infectious Futures* short stories and the radio series *The Trouble With Hawaii* all enable encounters with individuals affected by AMR.

Many events delivered at festivals or other events by individual microbiology labs and their teams, or by sci–art collaborations, attract large audiences who are usually fascinated to learn about AMR and intrigued and stimulated by the diverse means used to engage. Our work in sourcing the activities described in this paper has been significant. We were impressed by the number and range of activities that were uncovered, but we were dismayed at the lack of citable information and the consequent loss of potential learning. It would be valuable if there were some central site, or interactive hub, to which such activities could be submitted, so that neither they nor the observations of the delivery teams are lost. This lack of citable material was a significant limitation to our study.

The first (‘a massive awareness campaign’) and second (‘improve hygiene and prevent the spread of infection’) commandments of the O’Neill report,^50^^,^^51^ and indeed the entire call to action, rely on good evaluation, emphasizing the importance of assessing the effectiveness of any AMR information intervention.^4^ Yet it is not easy to find information on the activities themselves (Table 1), let alone their success in terms of achieving aims. If the activities are not anchored in some sort of retrievable output, be it a website, magazine article or peer-reviewed paper, then they are easily lost (or never found) and lessons learned are not shared. Perhaps the delivery of the event itself is deemed sufficient, or its small contribution to overall public awareness is so insignificant, that evaluation becomes unnecessary or irrelevant. Does it matter that 37 adults met in a tunnel to learn about AMR and were inspired by the event?^37^ How did they feel about it 12 months later? Or is the continuous and diverse spreading of the message the best route to a science-literate society? For the scientist to answer these questions, they must utilize the common rules of the scientific method: have you achieved your aims/satisfied your objectives? No matter how small the event, its effectiveness or shortcomings ought to be clear.

The larger national initiatives were developed to address key messages for their audiences and they do include significant evaluations. They also provide resources to support those who are delivering events as well as good models for those who wish to develop their own resources and activities. It is also relatively easy to obtain/analyse data that demonstrate effectiveness of initiatives delivered through digital and social media. Markers of success do reveal knowledge and behavioural changes that contribute to the overall ‘fight against AMR’. It is important that we, as ‘experts’, deliver effective activities and ensure that they are shared with others and that they are critically evaluated to ensure maximum effectiveness and impact. It has been noted that there is an urgent need for microbial literacy in society^52^ and AMR provides a perfect example of this urgency. A central hub, through which activities designed to raise awareness of AMR could be disseminated, would greatly facilitate our efforts in this arena.

## Transparency declarations

None to declare.

## Supplementary Material

dlaa012_Supplementary_Data
