# Supplementary material for: Raising awareness of antimicrobial resistance among the general public in the UK: the role of public engagement activities
Source: JAC Antimicrob Resist. 2020 Mar 10;2(1):dlaa012. doi: 10.1093/jacamr/dlaa012 (PMC8210175; doi:10.1093/jacamr/dlaa012)
Supplement: dlaa012_Supplementary_Data [file dlaa012_supplementary_data.docx]

**Reviewer report 1 (R0 – review of original submission)**

**PLEASE NOTE: JAC-AMR aims to publish your anonymous peer review comments with the final article for transparency. Please indicate in the box below whether you give your consent for us to publish your peer review comments for this article.**

Yes

**Synopsis**

**1. If the article contains original data, does the synopsis adequately reflect the manuscript as a whole, with the most important aspects described (of the introduction, methods and key results, and a balanced conclusion)? If NO, what is required to improve the synopsis? Please use the box below.**

Not applicable

Lines 14-16 states that few events are documented which begs the question how did the authors find the information for the paper.
The suggestion of an information hub is not discussed within the article but appears as a key conclusion in the abstract.

**Introduction**

**2. Does the introduction provide the necessary background information for readers to understand the article? (e.g. does it describe the problem, current state of knowledge, etc.) If NO, what is required to improve it? Please use the box below**

No

**3. Is the research hypothesis and/or aims/objectives of the study clearly stated in the introduction? If NO, what is required to improve it? Please use the box below**

No

**4. Is there superfluous/unnecessary text in the introduction? (Only text that is required for readers to understand why the study has been done should be included). If YES, what text is superfluous and should be removed? Please use the box below**

No

Considering the answers above and any other comments/advice you may have, please indicate what is required to improve the Introduction:

The introduction is sparse and does not give much background on engaging the public. There are a wealth of studies that could be quoted from across Europe as well as the NICE guideline referred to later in the text which summarised the evidence in this area.
The objectives of this review of resources are not clearly stated in the introduction.
The sentence from lines 24-27 does not make sense.

**Methods**

**5. Were the methods used suitably robust and appropriate for the research question(s) and/or the aims/objectives of the study? If NO, the article should be rejected, but please advise the authors accordingly in the free-text box below and complete the review in full**

Not applicable

**6. Are the methods described in enough detail so the reader can understand what was done and interpret the results and conclusions appropriately?**

Not applicable

**7. Are the methods described in language that a reader who does not specialize in the area of the study/methods used will understand? For example, are the statistical approaches used described in language that non-statistician healthcare professionals will understand? If NO, please indicate what is required to improve the language in the methods section in the box below**

Not applicable

Considering the above and any other comments/advice you may have, please indicate other aspects of the methods section that could be improved in the free-text box below)

There is no methods section but in common with publications on review of the literature it would be helpful to include a description of methods used to identify the public engagement activities described  e.g. websites, databases used.
Currently the reader has no idea how the authors sourced the content.

**Results**

**8. Given the methods used, are results described accurately, in enough detail and in an appropriate format to adequately inform the discussion section and the authors/readers conclusions? If NO, please indicate what is required to improve the results section in the box below**

No

**9. Based on what is described in the methods section, are data/results (that are likely to be available to the authors) missing from the current draft that you think should be presented to improve the manuscript?**

Yes

**10. Are Tables/Figures clear, required and do they add/complement the results text (they should not simply repeat what is in the text or vice versa)? If NO, please indicate what is required to improve the Tables/Figures in the box below**

No

Considering the above and any other comments/advice you may have, please indicate what is required to improve the results section:

The manuscript is not structured with a results section but much of the content is discussion of the results of activities that have been documented.
One specific section is not required as it refers to activities of healthcare organisations rather than the public (lines 52-61)
The data quoted for Antibiotic Guardian and for social media is quite dated and I would suggest that more recent information is used as this is an area that has evolved significantly over the past few years.
Line 112 states that e-bug is led by PHE.- should clarify that PHE lead it in the UK but it is a Europe-wide resource.
The section Performance includes drama/music plus TV and radio - should these be separate sections?

Tables/Figures
I think Table 1 and Figure 1 are unnecessary and could be covered using text.
Table 3 refers to a SCOPUS document search tool - this not mentioned anywhere in the text but I assume it is a Method used?
A table to summarise all activities by category may be a useful addition to avoid it being text heavy.

**Discussion**

**11. Are the discussion and any conclusion(s) reasonable given the stated hypothesis/aims/objectives and results? If NO, please indicate how to improve the discussion in the box below**

No

**12. Is there superfluous/unnecessary text in the discussion? (Only text that directly relates to the results and understanding thereof in the context of existing published literature and the limitations of the study should be included.) If YES, please indicate what should be removed to improve the discussion in the box below**

No

**13. Is the Limitations sub-section adequate and reflective of the actual limitations of the study? ? If NO, please indicate how to improve the Limitations section in the box below**

No

Considering the above and any other comments/advice you may have, please indicate how to improve the discussion section:

The Abstract has a conclusion about having a hub for logging activities but this is not mentioned anywhere in the Concluding comments section.
A final statement of the range of activities, the lack of outcome measures and suggestions to improve resources, perhaps via an information hub, to support activities would be helpful.

No limitations are mentioned. Something about search engines used, exclusions, unpublished reports should be included.

**General**

**14. Is referencing throughout the manuscript satisfactory? (for instance are there statements for which you believe a supporting reference should be cited?) If NO, please indicate how to improve referencing in the box below**

Yes

**15. Is the language used (i.e. spelling, grammar, etc.) of high enough quality to be published as is? If NO, please indicate how to improve the language within the article in the box below**

Yes

**16. Are ethics/other approvals appropriate/adequate and satisfactorily described? ? If NO, please indicate how to improve this in the box below**

Not applicable

Considering the above and any other comments/advice you may have, please advise the authors how to improve these aspects of the article.

**Opinion and review articles only**

**17. Please advise the authors in the box below on how to improve their opinion or review article:**

Details have been included in the previous sections

|  **Recommendation** | |
| --- | --- |
|  | Accept (as is with no revisions) |
|  | Minor revision (with anticipation of subsequent acceptance if revisions are adequate) |
|  | Major Revision |
|  | Reject |

| **Would you be willing to review a revision of this manuscript?** | |
| --- | --- |
|  | Yes |
|  | No |

**Comments**

 Comments to the Author

If you have any additional comments to the authors about any aspect of the manuscript not covered in the structured review above, please place these in the box below. If you have no additional comments, please say “No additional comments” in box.

No additional comments
